# In Vivo Efficacy of a Cocktail of Human Monoclonal Antibodies (CL184) Against Diverse North American Bat Rabies Virus Variants

**DOI:** 10.3390/tropicalmed2030048

**Published:** 2017-09-20

**Authors:** Richard Franka, William C. Carson, James A. Ellison, Steven T. Taylor, Todd G. Smith, Natalia A. Kuzmina, Ivan V. Kuzmin, Wilfred E. Marissen, Charles E. Rupprecht

**Affiliations:** 1 Centers for Disease Control and Prevention, 1600 Clifton Rd, Atlanta, GA 30333, USA; ioy8@cdc.gov (W.C.C.); hio6@cdc.gov (J.A.E.); ire2@cdc.gov (T.G.S.); 2 East Tennessee State University, James H. Quillen College of Medicine, Johnson City, TN 37614, USA; staylor.trevor@gmail.com; 3 University of Texas Medical Branch, 301 University Blvd, Galveston, TX 50555, USA; natakuzmina@yandex.ru (N.A.K.); ivkuzmin@yandex.ru (I.V.K.); 4 Crucell Holland BV, Archimedesweg 4, 2333 CN Leiden, The Netherlands; wmarissen@hotmail.com; 5 LYSSA LLC, Cummings, GA 30040, USA; charles_rupprecht@yahoo.com

**Keywords:** bat viral diseases, monoclonal antibody, immune globulin, lyssavirus, post-exposure prophylaxis, rabies, vaccine, zoonosis

## Abstract

Following rabies virus (RABV) exposure, a combination of thorough wound washing, multiple-dose vaccine administration and the local infiltration of rabies immune globulin (RIG) are essential components of modern post-exposure prophylaxis (PEP). Although modern cell-culture-based rabies vaccines are increasingly used in many countries, RIG is much less available. The prohibitive cost of polyclonal serum RIG products has prompted a search for alternatives and design of anti-RABV monoclonal antibodies (MAbs) that can be manufactured on a large scale with a consistent potency and lower production costs. Robust in vitro neutralization activity has been demonstrated for the CL184 MAb cocktail, a 1:1 protein mixture of two human anti-RABV MAbs (CR57/CR4098), against a large panel of RABV isolates. In this study, we used a hamster model to evaluate the efficacy of experimental PEP against a lethal challenge. Various doses of CL184 and commercial rabies vaccine were assessed for the ability to protect against lethal infection with representatives of four distinct bat RABV lineages of public health relevance: silver-haired bat (Ln RABV); western canyon bat (Ph RABV); big brown bat (Ef-w1 RABV) and Mexican free-tailed bat RABV (Tb RABV). 42–100% of animals survived bat RABV infection when CL184 (in combination with the vaccine) was administered. A dose-response relationship was observed with decreasing doses of CL184 resulting in increasing mortality. Importantly, CL184 was highly effective in neutralizing and clearing Ph RABV in vivo, even though CR4098 does not neutralize this virus in vitro. By comparison, 19–95% survivorship was observed if human RIG (20 IU/kg) and vaccine were used following challenge with different bat viruses. Based on our results, CL184 represents an efficacious alternative for RIG. Both large-scale and lower cost production could ensure better availability and affordability of this critical life-saving biologic in rabies enzootic countries and as such, significantly contribute to the reduction of human rabies deaths globally.

## 1. Introduction

Rabies is an acute progressive encephalitis caused by lyssaviruses. Despite significant progress in our understanding of rabies pathobiology and epidemiology, and major advancements in the development of safe and effective biologics for disease prevention, this neglected zoonosis causes approximately 60,000 human deaths annually [1,2]. Although dogs are the major global reservoir for rabies virus (RABV), bats are responsible for the majority of human rabies fatalities in the Americas, Australia and Western and Central Europe. Regardless of the source of viral exposure, human rabies is preventable with proper wound care, prompt administration of modern vaccine and rabies immune globulin (RIG) [3,4]. Over the past several decades, post-exposure prophylaxis (PEP) schedules have evolved, encompassing fewer doses of both intramuscular (i.m.) as well as dose-sparing intradermal (i.d.) routes for administration of inactivated vaccine in as few as four doses. However, in the absence of licensed, commercially available, live-attenuated rabies vaccines, administration of RIG remains a critical component of PEP when inactivated vaccines are used [3,5].

Current commercially available human and equine RIGs (HRIG, ERIG) are produced via pooling of human or equine plasma from immunized donors. Such production processes are associated with significant costs as well as with a possibility for transmission of potential bloodborne pathogens. Low-scale manufacturing, coupled with prohibitive cost, renders these immune globulins virtually unavailable for a majority of the population at risk in rabies-enzootic countries such as Asia and Africa, where the demand is the highest. New approaches, such as the use of hybridoma and humanization technologies, as well as use of single chain and VHH single domain antibodies, allow for cell culture or microbial expression systems production of monoclonal antibodies (MAbs), a promising alternative to polyclonal RIG with reduced risks for transmission of pathogens and large-scale production for a reduced cost. An inherent disadvantage of any MAb, however, is the specificity/affinity to a single binding epitope on a viral protein and consequently a diminished breadth of neutralizing activity for certain virus variants with amino acid substitutions that prevent MAb binding [5,6].

The concept of using a cocktail of at least two MAbs, which target distinct, non-overlapping epitopes and that do not compete for binding to the RABV glycoprotein, as a potential alternative to RIG in PEP, has been widely accepted by the scientific community and also endorsed by WHO [3,7,8,9,10]. CL184 is a cocktail of two human MAbs (CR57 and CR4098), produced in human PER.C6^®^ cells. CL184 meets the criteria of binding to different epitopes (CR57 to epitope I, CR4098 to IIIa) and does not engender competition for the binding to RABV glycoprotein [8,11]. Previously, CR57, CR4098 and CL184 were evaluated in vitro against a panel of 26 distinct RABV isolates of public health and veterinary significance [12]. Although CR57 alone did not neutralize a south central skunk RABV and big brown bat RABV (*Eptesicus fuscus* western lineage 1, Ef-w1); and CR4098 alone did not neutralize mongoose RABV from South Africa, big brown bat RABV (*Eptesicus fuscus* eastern lineage 1, Ef–e1) or western canyon bat (*Parastrellus hesperus* from Arizona) [8], it was shown that the combination of these two MAbs, CL184, did provide neutralization of all 26 tested RABV isolates [12], as well as neutralization of an additional panel of 18 RABV isolates (reported in this manuscript, Table 1). Furthermore, it was shown retrospectively that the lack of neutralization was related to epitope mutations introduced during cell culture amplification of the primary RABV isolates in the case of the south-central skunk RABV and the western canyon bat (Ph) RABV from Arizona (previously unpublished results). During one in vivo experiment, CL184, in combination with vaccine, protected hamsters against a lethal challenge with canine RABV, when administered 24 h after exposure, which was comparable with the results obtained for HRIG. In addition, CL184 was similar to HRIG in demonstrating a lack of interaction with vaccine [12]. These results suggested that CL184 could be an efficacious alternative to RIG as a part of rabies PEP.

Given the public health importance and the diversity of bat RABV present in the Americas, as well as the frequency and distribution of isolates with mutations in the MAb binding epitopes [13], the objective of this study was to evaluate the efficacy of CL184 against selected distinct bat RABV variants from North America (including those having a critical mutation in the MAb-binding site on the viral glycoprotein). The work was done using an animal model to compare vaccine protection using standard PEP (that included HRIG and commercial rabies vaccines) against those using CL184 in substitution for HRIG.

## 2. Materials and Methods

### 2.1. Animals and Viruses

**Figure 1:**
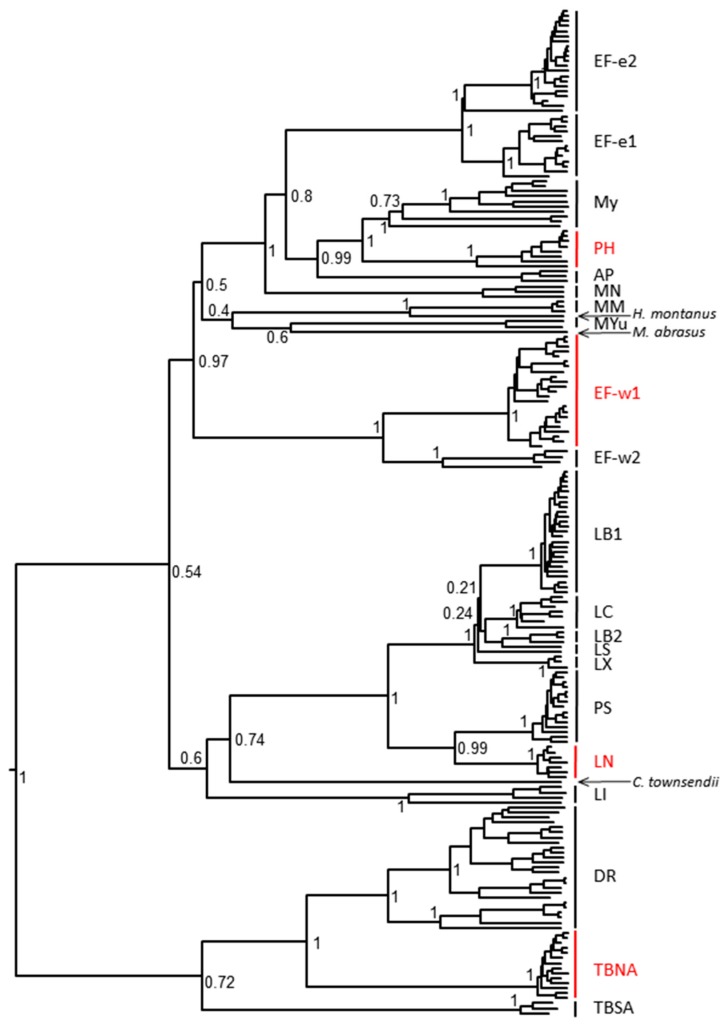
Phylogenetic relationship of rabies virus isolates used in this study with other representatives of bat RABV lineages. RABV used in this study are highlighted in red (PH—*Parastrellus hesperus*, Ef-w1—*E. fuscus* western lineage 1, LN—*Lasionycteris noctivagans*, TBNA—*Tadarida brasiliensis* North America).

Two-month-old female Syrian hamsters (*Mesocricetus auratus*), weighing approximately 100–120 g, were obtained from commercial suppliers and held for acclimation for 3–7 days upon arrival before use. Four different RABV isolates, representatives of distinct bat-associated RABV lineages (Figure 1), were used as a challenge in PEP experiments. An *Eptesicus fuscus* Ef-w1 RABV (A09-2400L), 10^6.1^ 50% mouse intracerebral lethal doses (MICLD_50_)/50 μL, was isolated from the salivary glands of a naturally infected gray fox (*Urocyon cinereoargenteus*) in Arizona. A *Parastrellus hesperus* RABV (Ph 3860 RABV, A07-0449), 10^4^ MICLD_50_/50 μL, was isolated from the salivary glands of a naturally infected western canyon bat from Arizona. A *Lasionycteris noctivigans* RABV (WA Ln RABV, A04-0723 and A12-6377), 10^6^ MICLD_50_/50 μL, was isolated from the salivary glands of a naturally infected silver-haired bat from Washington. A *Tadarida brasiliensis* RABV (Tb RABV, A14-3368 and TX3368), approximately 10^5^ TCID_50_/100 μL, was isolated from the brain of a naturally infected Mexican free-tailed bat from Texas. All the original isolates were amplified in cell culture or following i.c. challenge in mice. The titer of viruses was determined in mouse neuroblastoma cell culture and expressed in the 50% tissue culture infectious doses (TCID_50_) or focus forming units (FFU) as well as via titration in mice (MICLD_50_) and relative pathogenicity was determined in naive Syrian hamsters prior to experimental prophylaxis. Only RABV isolates that produced at least 75% mortality in this model were selected for further experiments with a sample size determined accordingly. All animal handling and experimental procedures were undertaken in compliance with CDC Institutional Animal Care and Use Committee guidelines (protocols #1593FRAHAMC and 2266FRAHAMC).

### 2.2. Biologics

A volume of 50 μL of the commercial inactivated human diploid cell vaccine (HDCV), Imovax^®^ (Sanofi Pasteur, Lyon, France) or purified chicken embryo cell vaccine (PCECV), RabAvert^®^ (Novartis Vaccines, Marburg, Germany; for Tb groups), with a minimum potency of 2.5 IU/mL was administered via the intramuscular (i.m.) route, according to the Essen (on days 0, 3, 7, 14 and 28) or modified Essen (on days 0, 3, 7 and 14) regimen. In addition, 50 μL (20 IU/kg) of HRIG (Imogam^®^ Rabies-HT (Sanofi Pasteur, 150 IU/mL)) or 6, 12, 16, 18 or 24 μg/kg of CL184 (mixture of CR57 and CR4098 in 1:1 protein ratio) was administered i.m. into the site of virus inoculation at day 0.

In all of our experiments, the amount of MAbs administered was expressed in μg/kg as opposed to IU/kg standardly indicated for polyclonal RIG products. HRIG is a polyclonal product consisting of many non-specific proteins with a very small fraction of rabies-specific antibodies, and hence correlation between protein concentration (μg/kg) and rabies virus specific neutralization (IU/kg) could not easily be established. In contrast, cell cultures producing only one anti-rabies MAb combined with protein purification techniques result in highly purified MAb devoid of other contaminants. Hence, such antibodies can be accurately quantified and thus dosed on basis of μg/kg, thereby excluding dosing variability as a result of inconsistency in potency measurements.

### 2.3. Experimental Design

The calculations of group sample sizes for each individual RABV isolate were based on statistical analysis and taking into account the mortality of naïve (non-treated) hamsters during preliminary experiments. To achieve the statistical power required to demonstrate the potential added benefit of tested biologics, we selected a cutoff in mortality of ≥75%. If mortality was 100% in RABV-challenged hamsters (via a titration experiment), a group size of 12 animals was considered adequate and selected for consequent experiments. If, however, the mortality of naïve animals was <100% but >75%, a group size of 21 animals was selected as adequate for comparative non-inferiority non-clinical experiments.

### 2.4. Model Validation/Post-Exposure Prophylaxis (PEP) Initiation Determination

Prior to the evaluation of PEP efficacy, determination of the PEP initiation window was conducted. Approximately two-month-old female Syrian hamsters (*n* = 12 or 21) were assigned randomly to experimental groups and infected into the left gastrocnemius muscle with an expected lethal dose of RABV (Ef-w1 RABV, Ph 3860, WA Ln, Tb RABV, based upon prior observations; unpublished data). Thereafter, PEP was initiated 2, 6 or 24 h following the challenge. On days 0 (set as the day of PEP initiation), 3, 7, 14, +/− 28 the animals received a dose of rabies vaccine (HDCV for Ef-w1, Ph and WA Ln; and PCECV for Tb) applied into the right gastrocnemius muscle. In addition, HRIG at a dose of 20 IU/kg was administered at the initiation of PEP into the same i.m. location as virus challenge. For comparison, besides a control (PBS only) group, a vaccine-only group was included with the same time windows of PEP initiation. Monitoring was the same as below for efficacy experiments.

### 2.5. Evaluation of the Efficacy of HRIG/Vaccine versus CL184/Vaccine during PEP

Female Syrian hamsters (*n* = 12 or 21) were assigned randomly to experimental groups and infected in the left gastrocnemius muscle with a lethal dose of RABV. Timeline for challenge and initiation of PEP, as well as viral dose used, were selected based on the model validation experiments and prior experimental data regarding particular virus pathobiology in hamster model. The PEP was initiated 24 h (Ln, Ph and Ef-w1 RABV) or 2 h post infection (p.i.) (Tb RABV). On days 0, 3, 7, 14, and 28 the animals received a dose of rabies vaccine. In addition, 50 μL of HRIG at 20 IU/kg or 50 μL of different doses of CL184 at (6, 12 or 16 μg/kg for Ln, Ph and Ef-w1 RABV or 12, 18 and 24 μg/kg for Tb RABV), were administered i.m. at the site of virus inoculation on day 0. The animals were followed for 45 days and their clinical signs were monitored. All animals developing any specific signs of rabies were euthanized immediately according to an IACUC approved clinical score. Brains were removed at necropsy and subjected to detection of rabies virus antigens by the direct fluorescent antibody (DFA) test, as described below. Similarly, all animals surviving at the end of the experimental period were euthanized and their brains examined for the presence of RABV antigens.

### 2.6. Laboratory Methods

#### 2.6.1. Direct Fluorescent Antibody (DFA) Test

The RABV antigens were detected in brain samples using the DFA test [14] with a fluorescein-isothiocyanate (FITC)-conjugated anti-RABV MAb (Fujirebio Diagnostics, Inc., Malvern, PA, USA).

#### 2.6.2. Rapid Fluorescent Focus Inhibition Test (RFFIT)

The rapid fluorescent focus inhibition test (RFFIT) was performed according to a standard, previously described protocol [15].

#### 2.6.3. Reverse Transcription Polymerase Chain Reaction (RT-PCR), Hemi-Nested RT-PCR (hnRT-PCR) and Sequencing

To confirm identity of RABV in central nervous system (CNS) tissue of euthanized animals with the initial inoculum and to identify any potential selection of escape mutations, total RNA was extracted from the CNS tissue samples using TRIZol reagent (Ambion, Carlsbad, CA, USA) according to the manufacturer’s recommendations. The RT-PCR was performed as described elsewhere [16]. The RT-PCR products were purified and subjected to direct sequencing on an ABI 3730 DNA Sequencer (Applied Biosystems, Carlsbad, CA, USA). The complete and partial nucleotide G gene sequences were assembled and converted into amino acid sequences using the Bio Edit program, v.7 (Ibis Biosciences, Carlsbad, CA, USA) [17]. Amino acid sequences of the aligned MAb binding epitopes were compared across the dataset.

### 2.7. Statistical Analysis

Kaplan–Meier survival curves were calculated in the Statistical Analysis Software (SAS, version 9.2. SAS Institute Inc., Cary, NC, USA). The log-rank test was used to test differences between group survival distributions. The null hypothesis of identical survival functions was rejected at *p* < 0.05. GraphPad Prism, version 7 (GraphPad Software, La Jolla, CA, USA) was used to create survival curve graphs.

## 3. Results

### 3.1. Neutralization of Selected RABV Isolates In Vitro

An additional set of 18 RABV isolates of public health importance from Africa, Asia and Americas were tested for neutralization to complement the initial panel of 26 RABV isolates [12]. Twelve of the isolates were tested with CR57, CR4098, and HRIG (Imogam), and all isolates were neutralized with the exception of one, South Africa mongoose RABV, which was not neutralized by CR4098 (Table 1). This RABV isolate contains a N336D mutation in its glycoprotein which explains the observed lack of neutralization (data not shown). Further, 16 of 18 RABV isolates were tested and were shown to be efficiently neutralized by CL184 (Table 1).

### 3.2. Model Validation/PEP Initiation Window

**Figure 2:**
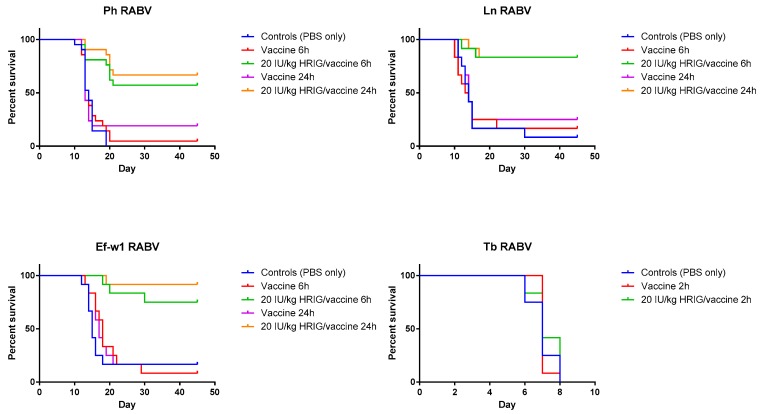
Validation of model and PEP initiation—Kaplan-Meier survival curves for Syrian hamsters after infection with bat rabies viruses. Hamsters (*n* = 12 or 21 per group) infected with the indicated RABV isolate 2, 6, or 24 h prior to intervention, received PEP consisting of either vaccine only (HDCV or PCECV) or vaccine in combination with 20 IU/kg HRIG.

In the experiments dedicated to the determination of PEP initiation window, survivorship of hamsters in control (placebo) groups was 8.3% for the Ln RABV, 0% for the Ph RABV, and 16.7% for the Ef-w1 RABV challenge. In contrast, in the vaccine-only group with PEP initiated 6 h p.i. the survivorship was 16.7%, 4.8%, 8.3%, whereas with PEP initiated 24 h p.i. it was 25%, 19%, 16.7% for these viruses, respectively (Table 2, Figure 2).

When HRIG + HDCV were administered 6 h p.i., 83.3%, 57% and 75% of experimental animals survived in the Ln RABV, Ph RABV, Ef-w1 RABV groups, respectively. Similarly, 83.3%, 66.7% and 91.7% of animals survived challenge with the Ln RABV, Ph RABV and Ef-w1 RABV, respectively, when HRIG and vaccine were administered 24 h p.i.

In the experiment with the Tb RABV, 0% survivorship was observed in the control as well as in the vaccine-only and in the HRIG + PCECV groups when biologics were administered 2 h p.i. (Table 2, Figure 2).

### 3.3. Evaluation of the Efficacy of HRIG/Vaccine versus CL184/Vaccine during PEP

**Figure 3:**
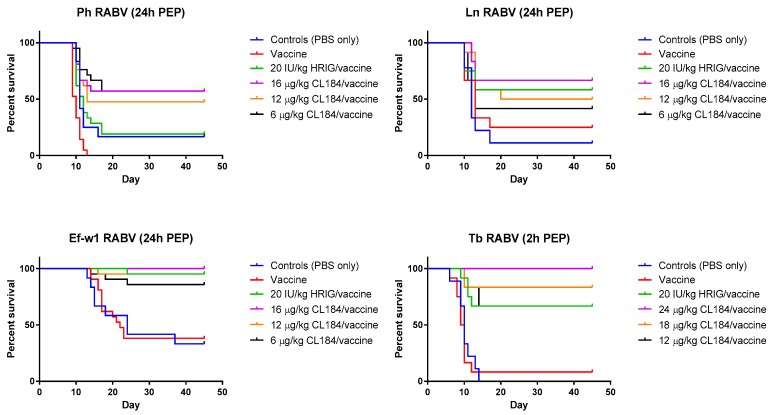
Evaluation of the efficacy of CL184/vaccine during PEP—Kaplan-Meier survival curves for Syrian hamsters after infection with bat rabies viruses. Hamsters (*n* = 12 or 21 per group) infected with a RABV isolate 2 or 24 h prior to intervention received PEP as outlined in Materials and Methods. Hamsters received 20 IU/kg HRIG or CL184 at a dosage of 6, 12 or 16 μg/kg for the Ln, Ph and Ef-w1 RABV or CL184 at a dosage of 12, 18 and 24 μg/kg for the Tb RABV.

In the experimental evaluation of the efficacy of biologics when administered 24 h p.i., Ln RABV, HRIG (20 IU/kg) + rabies vaccine (HDCV) resulted in 58% survival, whereas survival of animals in groups treated with 6 μg/kg, 12 μg/kg, or 16 μg/kg of CL184, 42%, 50% and 67% survived, respectively (Table 3, Figure 3). In a mock-control group and in the vaccine-only group, 11% and 25% of experimental animals, respectively, survived the challenge.

In the experiment where a Ph RABV isolate was used, administration of HRIG (20 IU/kg) + HDCV resulted in 19% survival whereas survival in groups treated with 6 μg/kg, 12 μg/kg and 16 μg/kg of CL184 was 57%, 48% and 57%, respectively (Table 4, Figure 3). In contrast, survivorship of 17% and 0% was observed in the mock-control and in the vaccine-only group, respectively, following Ph RABV challenge.

Combination of HRIG + HDCV resulted in 95% survivorship when administered 24 h p.i. with the Ef-w1 RABV variant, whereas administration of 6 μg/kg, 12 μg/kg and 16 μg/kg of CL184, in combination with HDCV, resulted in 86%, 95% and 100% survivorship, respectively (Figure 3). A survivorship of 33% and 38% was observed in the mock-control and in the vaccine-only groups, respectively, for this virus (Table 3, Figure 3).

When PEP was initiated 2 h p.i. with Tb RABV, 67% of animals survived in the HRIG + PCECV group, whereas administration of 12 μg/kg, 16 μg/kg and 24 μg/kg of CL184, in combination with PCECV resulted in 67%, 83% and 100% survivorship, respectively (Figure 3). A survivorship of 0% and 8% was observed in the mock-control and the vaccine-only group.

### 3.4. Sequence Analyses of the Original Inoculum and Virus Detected in CNS of Experimental Animals

When G nucleotide sequences of the original Ph 3860 isolate and its cell culture passages were compared to each other, it was confirmed that the first cell culture passage contained a mix of two variants, I338 and T338 (within the CR4098 binding epitope) and that consensus sequences of viral populations from further cell culture passages demonstrated solely the T338 variant. Virus recovered from the infected hamsters (following experimental challenge) resulted in a detection of either I338, or T338, or both phenotypes irrespective of biologics used in PEP and in the mock-control groups. All other output viruses matched the input virus demonstrating CL184 did not select for escape mutations. Phylogenetic relationship of Ph 3860 as well as other bat RABV isolates used in this study to other relevant bat RABV viruses is depicted in Figure 1 and relevant epitopes for virus variants used in this experiment are shown in Table 4.

## 4. Discussion and Conclusions

The scarcity of conventional RIG prompted research and development of alternatives. Multiple MAbs and their combinations have been evaluated in vitro and in vivo during the past decade as potential replacements for RIG (e.g., SO57 [7]; CR57, CR4098, [8,9,12]; RAB1 [18]; E559.9.14, 1112-1, 62-71-3, M727-5-1, and M777-16-3 [19]; RVC20-RVC58 [3,10,20]).

A common denominator for all individual MAbs is their limited breadth of neutralization, inevitably resulting in the inability of one MAb to neutralize the entire spectrum of RABV variants. However, as previous in vivo experiments of [8,11] have demonstrated, this can be compensated by a combination of two MAbs, which bind to non-overlapping epitopes.

Our study has shown that in cases of severe exposures to bat RABV (i.e., high virus doses delivered intramuscularly), administration of either HRIG or CL184, is critical for rapid peripheral neutralization and clearance of rabies virus. In both mock-control (placebo) and inactivated rabies vaccine-only groups, the mortality of 62–100% was observed (Table 3 and Table 4). In contrast, CL184, when administered in a dose ≥6 μg/kg in combination with vaccine, provided a significant benefit compared to vaccine alone.

In addition, the efficacy of CL184 plus vaccine in a dose ≥12 μg/kg was not inferior when compared to PEP consisting of HRIG (20 IU/kg) and rabies vaccine (HDCV), with 50–67% of experimental animals surviving a Ln RABV challenge, 48–57% surviving Ph RABV challenge, 95–100% surviving Ef-w1 challenge, and 67–100% surviving a Tb RABV challenge (Table 4, Figure 3).

Importantly, our experiments have shown that CL184 is efficacious against challenge with Ph RABV, which was not neutralized in vivo by the MAb CR4098, a component of the CL184 cocktail, given the mutation I338T (Table 1, Table 2 and Table 4; Figure 3). Of note, this mutation was introduced during cell culture amplification of the primary Ph RABV isolate as indicated by G gene sequencing of a series of virus stocks. Epitope mutations might result either from adaptation of the primary RABV isolates to cell culture as shown in the case of south central skunk RABV (Rupprecht, Marissen, personal communication). Alternatively, both sequence variants might be present in the original field isolate in different proportions. Although initially described as a result of selection of CVS rabies strain mutants following culture with neutralizing anti-glycoprotein antibodies [21], our study demonstrated that the I338T mutation affecting neutralization could also occur as a result of amplification in cell culture or in the laboratory rodent model without antibody-mediated selection pressure. Of note, 338T did not appear in consensus sequences of 10 natural Ph RABV isolates (data not shown). The Ph 3860 virus with the predominant I338T substitution served as a good model to assess the in vivo efficacy of CL184 given that it was not neutralized in vitro by one of the cocktail MAbs.

Natural occurrence of RABV with mutation(s) in the MAb binding epitope is, however, critical for the assessment of the adequacy of a particular MAb or cocktail of MAbs to be used as PEP in a particular geographic area. Sequencing of epitopes has been shown to be a reliable predictor of MAb neutralization capacity in vitro and in vivo. As previously described [13], the binding epitope for CR57 is relatively conserved with only one isolate/sequence exception (frequency 0.1%; 1/1042, Chinese dog, SE Asia-2), the K226M substitution, shown to preclude binding (data not shown). However, this reported mutation is more likely to be a sequence error rather than representing a true natural isolate, as in more than 175 Chinese RABV isolates no critical mutation in the CR57 epitope was observed (data not shown). Although the frequency of substitutions precluding neutralization of MAb CR4098 in antigenic site III is higher (N336D, 63/1042, 6%, including besides others big brown bat (*Eptesicus fuscus*) RABV from North America), our study showed that combining CR57 and CR4098 in a cocktail can effectively neutralize virus in vivo even if one of those MAbs does not neutralize it in vitro. Of note, CR4098 was still shown to bind to RABV glycoprotein harboring an N336D mutation [8] which could facilitate viral clearance in vivo. In addition, in neutralization experiments using a natural big brown bat (*Eptesicus fuscus*) RABV isolate harboring a N336D mutation, complete neutralization by CR4098 at 15 µg/mL was observed (data not shown). Overall, these findings emphasize the importance of WHO recommendations requiring inclusion of at least two MAbs [3] with non-overlapping epitopes in biologics for PEP as well as a need for continuous surveillance for natural occurrence of RABV isolates with mutations which may preclude MAb binding.

Although effective concentration of immune globulin in the circulation of individual experimental animals was not measured during the observation period, mortality and survivorship data demonstrated a dose effect, with lower Mab doses resulting in higher mortality.

Virulence of different RABV variants influences the efficacy of PEP. As demonstrated in this study, viruses which are more pathogenic in a particular model (e.g., Tb RABV) and possibly spread more rapidly towards the CNS, require an earlier initiation of PEP (2 hours p.i.) as compared to other viruses, for which PEP initiated 6 or 24 h p.i. still seemed to provide an adequate prophylactic effect within the hamster model (Table 4). Although pathogenesis is dependent on route, viral dose, host species and proximity the exposure site to the CNS, further studies may elucidate differences in the kinetics of peripheral neuronal entry and axonal spread of various RABVs.

In this study, the efficacy of CL184, when administered in a dose ≥12 μg/kg in combination with a commercial inactivated rabies vaccine, was not inferior to PEP consisting of HRIG and the same vaccine. As such, CL184 presents a promising, non-inferior alternative for RIG during rabies PEP. Large scale and lower cost production of MAbs could ensure availability and affordability of this critical life-saving biologic in rabies enzootic countries and would significantly contribute to the reduction of human rabies deaths globally.

## Figures and Tables

**Table 1:** Breadth of in vitro neutralization of HRIG, CL184 and its components against selected RABV isolates not covered by previous publications [12].

Lyssaviruses	HRIG *	CR57	CR4098	CL184
Cow/dog, Sri Lanka	+	+	+	+
Dog, China 2005	+	+	+	+
Dog, China (RV342)	+	+	+	+
Dog, India (I 148)	NT	NT	NT	+
Dog, India (I 151)	+	+	+	+
Dog, India (I 155)	+	+	+	NT
Dog, Philippines	+	+	+	+
Dog, Philippines (231/002)	+	+	+	NT
Dog, Tunisia	+	+	+	+
Human/dog, UK ex India	NT	NT	NT	+
Human/wolf, Russia Siberia (RVHN)	+	+	+	+
Mongoose, South Africa	+	+	-	+
Raccoon dog, Russia/Far East	+	+	+	+
Skunk, south central (SK4384)	+	+	+	+
Bat, *Lasiurus borealis*, TN (tn132)	NT	NT	NT	+
Bat, *Lasiurus borealis*, TN (tn269)	NT	NT	NT	+
Bat, *Lasiurus borealis*, VA (VA399)	NT	NT	NT	+
Bat, *Lasiurus cinereus*, TN	NT	NT	NT	+

* Imogam (Sanofi Pasteur); + indicates neutralization; NT—not tested.

**Table 2:** Validation of animal model and postexposure prophylaxis (PEP) initiation (*p*-values based on log-rank Mantel-Cox test, comparing CL184 with standard PEP regimen 20IU/kg HRIG/vaccine).

	Survival after 45 days observation							
Group	Ln (%)	*p*-Value *	Ph (%)	*p*-Value	Ef-w1 (%)	*p*-Value	Tb (%) ^‡^	*p*-Value
**Control (PBS only)**	8.3	-	0	-	16.7	-	0	-
**Vaccine only, 6 h p.i.**	16.7	0.9984	4.8	0.5224	8.3	0.0023	0	0.8619
**Vaccine only, 24 h p.i.**	25	0.5174	19	0.6200	16.7	0.3391	NA	
**HRIG/vaccine, 6 h p.i.**	83.3	0.0002 0.0007	57	<0.0001 <0.0001	75	0.0005 0.0002	0	0.3901 0.3161
**HRIG/vaccine, 24 h p.i.**	83.3	<0.0001 0.0023	66.7	<0.0001 0.0002	91.7	<0.0001 0.0001	NA	-

* *p*-Value for vaccine-only group is comparison to control; HRIG/vaccine groups, first *p*-value is comparison to control group and second is comparison to vaccine-only group. (Ph—*Parastrellus hesperus*, Ef-w1—*Eptesicus fuscus* western lineage 1, Ln—*Lasionycteris noctivagans*, Tb—*Tadarida brasiliensis* North America). ^‡^ Groups challenged with Tb RABV were administered PEP at 2 h p.i. with PCECV vaccine.

**Table 3:** Evaluation of the efficacy of CL184/vaccine during PEP (*p*-values based on log-rank Mantel-Cox test, comparing CL184 with standard PEP regimen 20 IU/kg HRIG/vaccine).

	Survival after 45 Days Observation							
Groups	Ln (%)	*p*-Value *	Ph (%)	*p*-Value	Ef-w1 (%)	*p*-Value	Tb ^‡^ (%)	*p*-Value
**Control (PBS only)**	11.1	-	16.7	-	33.3	-	0	-
**Vaccine only**	25	0.3630	0	0.0034	38.1	0.8464	8.3	0.6668
**20 IU/kg HRIG/vaccine**	58.3	0.0430 0.1477	19	0.8705 0.0003	95.2	<0.0001 <0.0001	66.7	0.0007 0.0006
**24 μg/kg CL184/vaccine**	NA	-	NA	-	NA	-	100	0.0319
**18 μg/kg CL184/vaccine**	NA	-	NA	-	NA	-	83.3	0.3959
**16 μg/kg CL184/ vaccine**	66.7	0.5951	57.1	0.0177	100	0.3173	NA	-
**12 μg/kg CL184/ vaccine**	50	0.8931	47.6	0.0699	95.2	0.9862	66.7	0.9483
**6 μg/kg CL184/ vaccine**	41.7	0.5257	57.1	0.0062	85.7	0.2847	NA	-

* *p*-Value for vaccine-only group is comparison to control; HRIG/vaccine groups, first *p*-value is comparison to control group and second is comparison to vaccine-only group; CL184/vaccine groups is comparison to HRIG/vaccine group. (Ph—*Parastrellus hesperus*, Ef-w1—*Eptesicus fuscus* western lineage 1, Ln—*Lasionycteris noctivagans*, Tb—*Tadarida brasiliensis* North America). ^‡^ PCECV vaccine was used for Tb group while HDCV for Ef-w1, Ph and WA Ln.

**Table 4:** Bat RABV isolates used in the animal studies.

RABV Isolate	CR57 Epitope (226–231)	CR4098 Epitope (330–338)	CR57 Neutralization	CR4098 Neutralization
Bat, *Lasionycteris noctivagans*	KLCGVP	KSVRTWNEV	Yes	Yes
Bat, *Parastrellus hesperus*	KLCGVP	KSVRTWNET *	Yes	No
Bat, *Eptesicus fuscus* w1 lineage ^§^	KLCGVP	KSIRTWNEI ^‡^	Yes	Yes
Bat, *Tadarida brasiliensis*	KLCGVS	KSVRTWNEI	Yes	Yes

* Ph 3860 RABV isolate used in our study has I338T mutation in CR4098 epitope precluding its neutralization by that particular MAb. We used this mutation (resulting from cell culture passage) as a model to test CL184 in vivo against virus which is not neutralized in vitro by one MAb from the cocktail. ^‡^ Although some naturally-occurring *Eptesicus fuscus* Ef isolates have N336D mutation in the antigenic site III precluding neutralization of CR4098, we have not had that particular isolate available for in vivo experiments. Our isolate with N336 was neutralized by both CR57 and CR4098. ^§^ Isolated from gray fox, *Urocyon cinereoargenteus*.
